# Effects of Bahir Dar Textile Factory Effluents on the Water Quality of the Head Waters of Blue Nile River, Ethiopia

**DOI:** 10.1155/2015/905247

**Published:** 2015-11-24

**Authors:** Abrehet Kahsay Mehari, Shewit Gebremedhin, Belayneh Ayele

**Affiliations:** ^1^Fisheries, Wetlands and Wildlife Management Department, Bahir Dar University, P.O. Box 5501, Bahir Dar, Ethiopia; ^2^Natural Resource Management Department, Bahir Dar University, P.O. Box 5501, Bahir Dar, Ethiopia

## Abstract

The study was conducted in 2013/14 with the objective of determining the effects of Bahir Dar textile factory effluents on the head of Blue Nile River water quality. Dissolve oxygen was higher at the upstream site of the river, whereas BOD5, TDS, and total alkalinity values were higher at wastewater outlet of the factory site. The mean values of dissolved oxygen, BOD5, and total alkalinity were above maximum permissible limits set by WHO for drinking water at head of Blue Nile River. The mean value of BOD5 was above permissible limit of IFC for textile effluents to be discharged to surface water. A total of 836 aquatic macroinvertebrate individuals belonging to 21 families were collected. The Shannon-Wiener Diversity Index, the Hilsenhoff family-level biotic index, family richness, and percent dipterans were calculated. Hilsenhoff family-level biotic index and percent dipterans metrics differed significantly among sampling sites (*P* < 0.05). Hilsenhoff family-level biotic index was higher at the most downstream site but percent dipterans were higher at site of discharge of effluent to the head of Blue Nile River. Therefore, there is indication that effluent demands frequent control and proper treatment before being discharged to the environment.

## 1. Introduction

Pollution of natural waters with waste water arising from various industries has become a serious problem globally. Textile industries are large industrial consumers of waters as well as producers of wastewaters with the increased demand for textile products leading to increase in the generation of textile wastewater, which makes the textile industry one of the main sources of severe pollution problems worldwide [1–3].

Effluents from the textile factory commonly contain high concentrations of organic and inorganic chemicals and are characterized by high Chemical Oxygen Demand (COD), Biological Oxygen Demand (BOD), Total Dissolved Solids (TDS), pH, Total Suspended Solids (TSS) values, and low dissolved oxygen (DO) value as well as strong color. The major concern with color is its aesthetic character at the point of discharge with respect to the visibility of the receiving waters [4–6].

The textile factory in Ethiopia dates back to 1939 in relation with Italian colonialism era, when the first industrial textile factory was established in Dire-Dawa in the name of Dire-Dawa textile mill. Since 2010, the Ethiopian government has put effort to improve, support, and expand the textile industry, serving the domestic market but mainly with the aim to export and be competitive at the global market [7]. Ethiopia has potential of building a textile factory with governmental support, offering low-cost production and raw material and with a growing young population eager for jobs [8]. The factory is one of the largest employers in Ethiopia, with 35,000 direct employees (cotton farming (10%) and textile/garment manufacturing (90%)), excluding the 500,000 engaged in the informal hand-loom weaving sector [9].

Bahir Dar textile factory is one of Ethiopia textile factories, manufacturing 100% cotton products, including yarns and fabrics. It was established in 1961 from the fund of Italian war reparation in the town of Bahir Dar, 570 km northwest of Addis Ababa, Ethiopia. The factory has production capacity of 17 tons per day of yarns and 5,000 meters' wool fabrics [10]. The major factories in Bahir Dar town including Bahir Dar textile factory, Bahir Dar tanneries, and Bahir Dar Abattoir are built at the edge of head of Blue Nile River, where most of them dispose their solid and liquid wastes directly into this river. Despite the extensive work that has been conducted on Bahir Dar tanneries effluents with regard to physicochemical parameter and aquatic macroinvertebrates [11], little information is documented about the effect of Bahir Dar textile factory effluents on head of Blue Nile River.

Therefore, the objective of this study was to investigate the effect of Bahir Dar textile factory effluents on the head of Blue Nile River using aquatic macroinvertebrate as bioindicators through addressing the following research questions: (1) What is the pollution level of textile factory effluents? (2) Do the observed water quality parameters in the study area exceed the maximum permissible limits of national and international standards? (3) To what extent are the water quality parameters higher than the standard? (4) To what extent is the headwater of the Blue Nile River affected by the effluents from the factory?

## 2. Materials and Methods

### 2.1. Study Areas

Bahir Dar, the capital of Amhara National Regional State, is situated on the southern shore of Lake Tana, the source of Blue Nile River, approximately 565 kms northwest of Addis Ababa at an altitude of 1801 m.a.s.l, having latitude of 11038′′N and longitude of 37010′′E. The average elevation in the town is about 1795 m.a.s.l with “Woina Dega” type of agroecological zone. The town covers an area of about 16,000 hectares. The study area experiences average annual rainfall that ranges from 1200 to 1600 mm and it has mean annual temperature of 26°C [15]. It is a rapidly expanding town with commercial centers, small industries, and residences in all sectors of the town. The textile factory located at the edge of head of Blue Nile River discharges its effluents directly into head of Blue Nile River. However, the water from this river is used for different purposes like drinking, livestock watering, hygiene, and irrigation by the downstream communities.

### 2.2. Sampling

**Figure 1:**
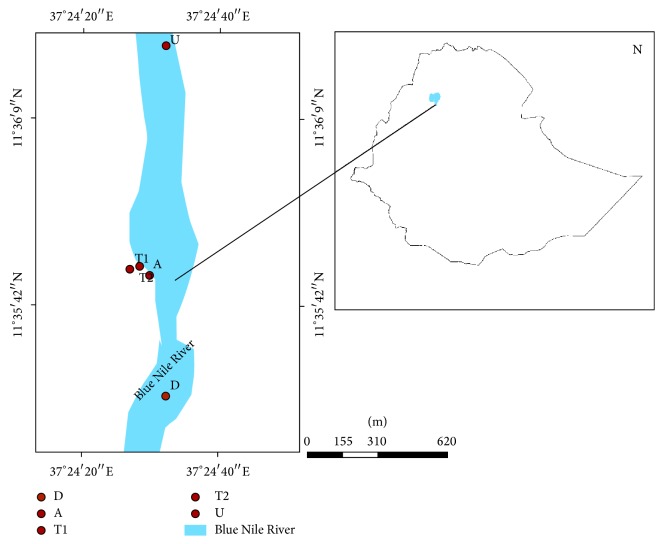
Location map of study area.

Five sampling sites were established on study area according to the method stated in [16]. The most upstream site from the head of Blue Nile River (U), the wastewater outlet of the factory (T1), neutralization pond (T2), where effluent is discharged and joins the head of Blue Nile River (A), and 200 m downstream site of waste joining the river (D) were chosen (Figure 1). Samples for both water quality parameters and aquatic macroinvertebrates were collected in August and December, 2013, and April of 2014. Samples for aquatic macroinvertebrates were not recorded from sampling sites T1 and T2 because these sites were concrete made ponds.

### 2.3. Data Collection

#### 2.3.1. Water Quality Parameters

Samples of water quality parameters, water temperature, dissolved oxygen (DO), pH, Total Dissolved Solids (TDS), and conductivity from all sites, were measured in situ using YSI 556 MPS multiprobe field meter. Water sample for BOD5, total alkalinity, and total hardiness were collected and stored in clean polythene bottles that had been prewashed with 10% nitric acid and thoroughly rinsed with deionized water using standard methods stated in [17]. Water samples for BOD5 were analyzed according to the standard methods of [18] at the laboratory of the Institute of Technology of Bahir Dar University, while samples for total hardiness and total alkalinity were analyzed at Amhara Design and Supervision Work Enterprise Laboratory using Paqualab photometer instrument with their respective palintest tablets.

#### 2.3.2. Aquatic Macroinvertebrate

Samples of aquatic macroinvertebrates were taken using *D*-frame dip net with mesh size of 500 *μ*m from U, A, and D sampling sites. In the field, those visible organisms were removed with forceps and put into the specimen bottles. All samples were preserved with 70% ethanol until laboratory analysis and counting. All the organisms in the sample were enumerated and identified to the family level using a dissecting microscope and standard keys [19–22].

#### 2.3.3. Data Analysis

Descriptive statistics were used to analyze the mean value and standard error of the water quality data. Four indices of the aquatic macroinvertebrate communities were calculated for each sampling site. The Shannon-Wiener Diversity Index (*H*′) is a diversity index that incorporates richness and evenness. A high *H*′ indicates a good water quality. *H*′ was calculated as follows:

(1) $$\begin{array}{c} H^{\prime }=-\sum \left(\;P_{i}\ln\left[\;P_{i}\;\right]\;\right), \end{array}$$

where *P*_*i*_ is the relative abundance (*n*_*i*_/*N*) of family *i*, *n*_*i*_ is the number of individuals in family *i*, and *N* is the total number of individuals in all families. *H*′ is ranging from 0 for a community with a single family to over 7 for a very diverse community.

The Hilsenhoff family-level biotic index (HFBI) is a biotic index that is calculated by multiplying the number of individuals of each family by an assigned tolerance value for that family. Assigned tolerance values range from 0 to 10 for families and increase as water quality decreases [23, 24]. This index was calculated as follows:

(2) $$\begin{array}{c} HFBI=\frac{\Sigma \left[\left(TV_{i}\right)\left(n_{i}\right)\right]}{N}, \end{array}$$

where TV_*i*_ is tolerance value for family *i*, *n*_*i*_ is the number of individuals in family *i*, and *N* is the total number of individuals in the sample collection. High HFBI community values are an indication of organic pollution, while low values indicate good water quality.

Research has indicated that the percentage of dipterans tends to increase with a decrease in water quality; they become increasingly dominant in terms of percent taxonomic composition and relative abundance along a gradient of increasing enrichment for heavy metals concentration [25, 26]. This index was calculated as follows:

(3) %  Dipterans=100∗#  Individual  DipteransTotal  Individuals  in  sample.

Family richness reflects the health of the community as a measurement of the variety of families present. Richness generally increases with increasing water quality, habitat diversity, and habitat suitability. This index was calculated as follows:

(4) FR=#  the  number  of  different  family  of  animals  in  the  sample.

Excel spreadsheets and Statistical Package for Social Sciences Software (SPSS version 20) were used for the statistical analysis. One-way ANOVA was used to evaluate differences in water quality data and aquatic macroinvertebrate metrics among the sampling sites. Differences among means were tested using Tukey HSD.

## 3. Results and Discussions

### 3.1. Water Quality Parameters

The mean values of dissolved oxygen ranged from 3.7 mg/L at sampling site T1 to 7.8 mg/L at U and showed significant variation among sampling sites (*F* = 15.259, *P* = 0.000). The value at site U was significantly higher than that at the other sites (Table 1). Many studies also showed that dissolved oxygen level of textile effluents is low and varied from 0.42 to 4.60 mg/L with average value of 2.36 mg/L [27], 4.8 mg/L–8 mg/L [12], about 0.4 mg/L [28], and 0.28 mg/L–5.12 mg/L [29]. The mean values of dissolved oxygen at sampling sites from head of Blue Nile River (U and D) were not acceptable for drinking purpose [30].

The mean values of BOD5 which varied from 5.3 mg/L at sampling site U to 40.3 mg/L at site T1 showed significant variation among sampling sites (*F* = 95.767, *P* = 0.000). The value at sampling site T1 was higher than that at the other sites (Table 1). The high value of BOD5 at T1 could be due to higher content of organic load and the high levels of BOD5 are the indicators of the pollution strength of the waters [13, 14]. The mean value of BOD5 of the study at sampling sites from head of Blue Nile River (U and D) was found to be above the permissible limit set for drinking water [30]. The mean value of effluent joining the head of Blue Nile River (A) was within the range of acceptable levels of [31] for textile effluents to be discharged into inland surface water bodies but was above standard limits of [32].

The pH mean values ranged from 7 at A to 8.7 at T1. The mean values did not differ significantly among sampling sites (*F* = 2.367, *P* = 0.123). The temperature mean values ranged from 20.4°C at A to 25.2°C at T1 and values did not vary among sampling sites (*F* = 0.718, *P* = 0.599) (Table 1). The mean values of temperature and pH of the study were within [31] as well as [30] guideline values. Similar to present study, [33, 34] reported the mean pH value of textile effluents in the range of 6–9. The temperature of textile effluents was also reported in the range of 27–34°C [12, 35].

The mean values of TDS varied from 101.7 ppm at sampling site U to 612.3 ppm at T1 and showed significant variation (*F* = 60.039, *P* = 0.000), with the value at T1 being significantly higher than that at other sampling sites (Table 1). Studies of these authors [27, 36] reported TDS values of textile effluents as 860 mg/L and 1260 mg/L, respectively, which are much higher than the TDS values recorded in the present study. The mean values of TDS at sampling sites U and D were within standards of [30] since water with a TDS < 1200 mg/L generally had an acceptable taste for drinking purposes.

The mean value for conductivity varied from 141 *μ*S/cm at sampling site U to 1050 *μ*S/cm at sampling site T1. There was significant variation among sampling sites (*F* = 110.142, *P* = 0.000), with the value at T1 being significantly higher than that at other sampling sites (Table 1). The conductivity for textile effluent was recorded in the range of 3804–1704 *μ*S/cm [37]. The mean value of conductivity at sampling sites (U and D) was within the range of the standard limit of [30] which is in the range of 400–800 *μ*S/cm for drinking purposes.

The mean values of total hardiness varied from 84 mL/g at sampling site U to 117 mg/L at A with mean value of 100 mL/g. Total hardiness did not show significance among sampling sites (*F* = 0.733, *P* = 0.590) (Table 1). Based on the classification Khopkar [38], hardness of natural water was classified into five categories on the basis of total ion content: soft (0–40 mg/L), moderately hard (40–100 mg/L), hard (100–300 mg/L), very hard (300–500 mg/L), and extremely hard (500–1000 mg/L). Based on this classification, the head of Blue Nile River water can be put in the category of moderately hard at upstream site (U) and hard at the most downstream site (D). The water containing excess hardness is not desirable for drinking [39]. As compared to present study, some studies [40, 41] reported higher mean values for textile effluents (in the range of 470–1010 mg/L CaCO_3_) and one study [29] reported slightly lower mean values (in the range of 63–88 mg/L CaCO_3_) for textile effluents.

Total alkalinity mean values ranged from 91 mg/L at U to 247 mg/L at T1. The mean values differ significantly among sampling sites (*F* = 16.8, *P* = 0.00) (Table 1). The mean values at sampling sites U and D are found to be above the permissible limit set for drinking water <75 mg/L [30] and this could be due to the use of soaps by local people for bathing and washing clothes which was apparent activities at these sampling sites. Studies of [27, 35] reported similar findings about the high alkalinity mean value of textile effluents.

### 3.2. Bioindicators

#### 3.2.1. Aquatic Macroinvertebrate Taxa

**Figure 2:**
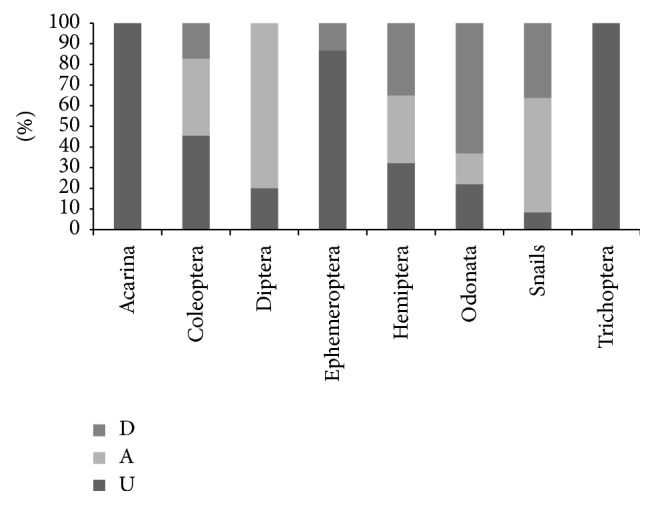
Relative abundance of main taxonomic group at each sampling site.

A total of 836 individuals composed of 21 families were collected from the three sites (U, A, and D). The total number of families present at each site ranged from 9 (D) to 11 (A). The most dominant families were Veliidae (103), Belostomatidae (102), Planorbidae (96), Gerridae (79), and Corixidae (64). The families least encountered were Tetragnathidae (4), Lestidae (4), and Aeshnidae (5). Furthermore, it is found out that more than 82.3% of the individuals collected belong to insect orders while less than 17.7% belong to the noninsect order like Acarina and Snail orders. Among the insect orders, Hemiptera and Coleopteran were the most dominant with 47.4% and 8%, respectively (Figure 2, Table 2).

Stated as in [26], sites that are with greater than 26 families are considered as nonimpacted, 19–26 as slightly impacted, 11–18 as moderately impacted, and 0-1 as severely impacted. Based on this finding, sampling sites U and D fall under moderately impacted while sampling site A fall under severely impacted site.

#### 3.2.2. Aquatic Macroinvertebrate Metrics

The mean value of Shannon-Wiener Diversity Index (*H*′) ranged from 2 at sampling site U to 1.8 at A indicating this site has less diversity than upstream and the most downstream sites (Table 3). The *H*′ value did not show significant variation among sampling sites (*F* = 1.00, *P* = 0.422). *H*′ is ranging from 0 for a community with a single family to over 7 for a very diverse community. *H*′ value of less than 1 indicates highly polluted, 1–3 moderately polluted, and greater than 4 unpolluted water bodies [42]. In present study, all the three sites are under moderate polluted conditions.

The mean values for Hilsenhoff family-level biotic index (HFBI) ranged from 6 at sampling site U to 7.9 at D. Mean values showed significant variation among sampling sites (*F* = 7.678, *P* = 0.022), with value being higher at the most downstream site (Table 3). This index was developed to detect organic pollution. In this study, all sites showed higher HFBI values, suggesting fair water quality conditions at sampling site U and poor at A and D.

The mean values for percent dipterans ranged from 0% at sampling site D to 10.7% at A and values showed significant variation among sampling sites (*F* = 9.494, *P* = 0.014), with mean value being high at site A (Table 3). Since most dipterans larvae contain hemoglobin, they are able to survive low oxygen conditions [43] and their higher abundance is indication of poor water quality [44].

## 4. Conclusion

We conclude that the textile factory poses serious pollution load to the aquatic habitat of the head of Blue Nile River which in turn makes the water highly polluted. The values of most metrics follow pollution gradient and showed deteriorated water quality conditions of the head of Blue Nile River. Therefore, hence, there are many immediate downstream users of the water river for drinking, fishing, bathing, and irrigation; the effluent demands frequent control and proper treatment before being discharged to the environment.

## Figures and Tables

**Table 1:** Mean values of water quality parameters of study sites compared to guideline values of WHO [12], EEPA [13], and IFC [14]. Different letters within the same row show significant variation among sampling sites according to Tukey HSD (*P* < 0.000).

Parameters	Sampling sites					Standards			
	T1	T2	A	D	U	*P* value	IFC	EEPA	WHO
DO (mg/L)	3.7b	7.7a	6.7a	7.2a	7.8a	0.000	NA	NA	>10
BOD5 (mg/L)	40.3a	36.3ab	33bc	8.2d	5.3df	0.000	30	50	<4
pH (pH Units)	8.4a	7.8a	7.1a	7.7a	7.3a	0.123	6–9	6–9	6.5–8.5
Temperature (°C)	25.2a	20.8a	20.4a	24.3a	23.6a	0.599	NA	40	<25
TDS (ppm)	612.3a	391b	447.8bc	129d	101.7df	0.000	NA	NA	<500
Conductivity (*μ*S/cm)	1050a	835.8b	943.7ab	168c	141cd	0.000	NA	NA	400–800
Total hardness (mg/L)	88a	102a	117a	110.33a	84a	0.590	NA	NA	NA
Total alkalinity (mg/L)	247a	225a	107b	94.67bc	91bcd	0.000	NA	NA	<75

NA: not available; IFC: International Finance Corporation; EEPA: Ethiopian Environmental Protection Authority; and WHO: World Heath Organization.

**Table 2:** Number of aquatic macroinvertebrates collected from study sites.

Family/order	TV	Sampling sites					
		T1	T2	U	A	D	Total
Acarina							
Tetragnathidae	4	NA	NA	4	0	0	4
Dipterans							
Ceratopogonidae	6	NA	NA	0	10	0	10
Chironomidae	8	NA	NA	9	0	0	9
Culicidae	8	NA	NA	0	30	0	30
Ephemeroptera							
Baetidae	5	NA	NA	48	0	3	51
Hemiptera							
Belostomatidae	9	NA	NA	37	43	22	102
Corixidae	8	NA	NA	0	35	29	64
Gerridae	9	NA	NA	0	68	11	79
Naucoridae	6	NA	NA	21	0	0	21
Notonectidae	9	NA	NA	0	22	5	27
Veliidae	7	NA	NA	94	8	1	103
Snails							
Hydrobiidae	8	NA	NA	0	25	8	33
Lymnaeidae	6	NA	NA	0	7	0	7
Physidae	7	NA	NA	0	8	0	8
Planorbidae	7	NA	NA	14	65	17	96
Odonata (Damsel and Dragon)							
Aeshnidae	3	NA	NA	5	0	0	5
Coenagrionidae	9	NA	NA	9	15	20	44
Cordulidae	3	NA	NA	15	0	0	15
Lestidae	9	NA	NA	0	4	0	4
Libellulidae	9	NA	NA	0	3	14	17
Trichoptera							
Hydropsychidae	4	NA	NA	40	0	0	40
Total individuals				328	373	134	835
Total families				11	14	9	21

TV = tolerance values, NA = not available.

**Table 3:** Mean values ± std. error of *H*′, HFBI, and % dipterans at each sampling site.

Sampling sites	*H*′	HFBI	% Dipterans
U	2 ± .00a	6 ± .14bc	2.7 ± 1.3b
A	1.8 ± .33a	7.5 ± .91ab	10.7 ± 3.5a
D	2 ± .11a	7.9 ± .26a	0 ± .00bc

^**∗**^Different letters within the same column show significant differences (*P* < 0.05). *H*′; Shannon-Wiener Diversity Index, HFBI: Hilsenhoff family-level biotic index, and Std. error; standard error.
